# Rich specialized insect damage on Pliocene leaves from the Mahuadanr Valley (India) growing under a warm climate with weak seasonality

**DOI:** 10.1002/ece3.11114

**Published:** 2024-03-11

**Authors:** Benjamin Adroit, Taposhi Hazra, Thomas Denk, Subhankar Kumar Sarkar, Mahasin Ali Khan

**Affiliations:** ^1^ Department of Palaeobiology Swedish Museum of Natural History Stockholm Sweden; ^2^ IMBE, Aix Marseille Univ, Avignon Univ, CNRS, IRD Marseille France; ^3^ Palaeobotany‐Palynology Laboratory, Department of Botany Sidho‐Kanho‐Birsha University Purulia India; ^4^ Entomology Laboratory, Department of Zoology University of Kalyani Kalyani, Nadia West Bengal India

**Keywords:** foliar damage, herbivory, Jharkhand India, leaf mining, plant–insect interaction

## Abstract

Plant‐insect interactions play a crucial role in shaping terrestrial ecosystems, influencing abundance and distribution of plant species. In the present study, we investigated leaf‐mining patterns on fossil leaves from Pliocene strata of the Mahuadanr Valley, Jharkhand, eastern India, deposited under a seasonal tropical climate, and reported complex interactions between plants and insects. We identified 11 distinct mining morphotypes. These morphotypes were mainly found on Dipterocarpaceae, Fabaceae, Lauraceae, and Moraceae; similar mining traces were also observed in the contemporary vegetation surrounding the fossil site. Although mining richness was relatively high, only 2.6% of all leaves in the fossil assemblage were mined. We compared mining richness and abundance values with previously reported values for galling. While richness was slightly lower for galling, almost 50% of all fossil leaves were galled. A literature survey on mining and galling patterns in modern vegetation suggests that there is no global explanation for richness of mining or gall‐inducing insects. Thus, low nutrient availability in the ancient forest, dominance of semideciduous leaves with hard texture, and different habitats in the same forest ecosystem, such as well‐drained forests and riparian stands, may all have favored different types of specialized plant–insect interactions.

## Graphical abstract

We investigated mining diversity in a Pliocene leaf assemblage of India and compared it to galling diversity; both functional feeding groups were relatively rich, but mining was much less abundant. Among possible factors determining the observed high galling intensity, we emphasized the role of semideciduous leaves with a hard texture, typical of tropical dry deciduous forests, as suitable hosts for gall‐inducing insects.

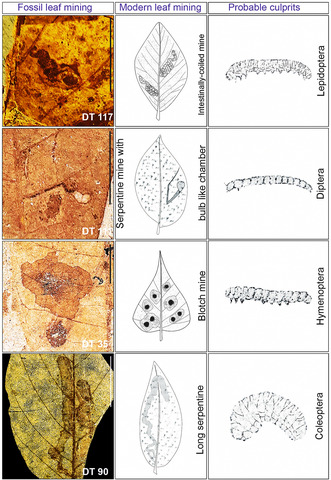

## 1 INTRODUCTION

Ecosystems are dynamic networks of biotic communities, where interactions with the abiotic environment are crucial for maintaining balance (Brooker & Callaghan, 1998; Ferranti et al., 2018; May, 1975; Tschirhart, 2000). Insects, through their interactions with plants, significantly affect plant and animal species distributions and ecological balance (Kamaru et al., 2024; Scudder, 2017; Strauss & Zangerl, 2002). Plant‐insect interactions, often antagonistic (Calatayud et al., 2018; Cariglino et al., 2021), are ubiquitous, varied, and influenced by morphological plant characteristics, plant species richness and composition, soil properties, and climate (Adroit et al., 2021; Calatayud et al., 2018; Júlião et al., 2014; Sinclair & Hughes, 2008).

The fossil record preserves these associations as damage traces on leaves, which have been categorized into different functional feeding groups in the guidebook ‘Guide to Insect (and Other) Damage Types in Compressed Plant Fossils’ (Labandeira et al., 2007). Richness and abundance of insect damage traces are well documented in fossil record (e.g., Adroit et al., 2018, 2021; Edirisooriya & Dharmagunawardhane, 2013; Grauvogel‐Stamm & Kelber, 1996; Hazra et al., 2022, 2023; Hickey & Hodges, 1975; Labandeira & Wappler, 2023; Larew, 1986; Schachat et al., 2014; Taylor & Scott, 1983; Wappler & Denk, 2011). The fossil record of insect herbivory on leaves traces the development of these interactions, and significant climatic episodes in the past were accompanied by marked changes in insect damage recorded on leaves. For instance, the Paleocene‐Eocene Thermal Maximum is characterized by an increase in insect damage on leaves (Currano et al., 2008). However, while these records capture snapshots of ecological dynamics, they do not always allow for direct quantitative climate reconstructions, as the same damage patterns can emerge under different environmental conditions (Labandeira & Wappler, 2023).

Among the different functional feeding groups, leaf mining is a particularly specialized habit, where endophagous insects consume live tissue from within the leaf blade, leaving the outer layers of the leaf partially intact, a feature that distinguishes it from other forms of herbivory (Connor & Taverner, 1997; Ding & Labandeira, 2014). Initiated by the oviposition of female phytophagous insects, the larvae of Diptera, Coleoptera, Hymenoptera, or Lepidoptera create distinctive tunnels or cavities within the leaf, known as leaf mining (Cariglino et al., 2021; Crane & Jarzembowski, 1980), which are indicative of the complex evolutionary interplay between plants and their insect herbivores (Connor & Taverner, 1997; Tooker & Giron, 2020). While the fossil record offers unique insights into ancient plant‐insect interactions, modern ecological studies do not reveal global patterns in the occurrences of these functional feeding groups, suggesting complex interplays influenced by local environmental factors (Blanche, 2000; Fernandes et al., 2004; Júlião et al., 2014; Sinclair & Hughes, 2008).

In the fossil record, leaf mining reflects biological activity related to the dietary niches of insects in deep time and hence provides a unique window into ecological and evolutionary associations of the past (Knecht et al., 2023; Labandeira et al., 2007; Winkler et al., 2010). The earliest unambiguous occurrence of leaf mining and galling, so far, dates back to the Cretaceous (Imada et al., 2022; Krassilov & Karasev, 2008; Labandeira, 2021; Labandeira & Anderson, 2005; Meller et al., 2011; Rozefelds & Sobbe, 1987; Scott et al., 1995, 2004) and records are increasing throughout the Mesozoic and Cenozoic (Donovan et al., 2014; Hickey & Hodges, 1975; Krassilov, 2007; Krassilov & Shuklina, 2008; Kuroko, 1987; Liebhold et al., 1982; Robledo et al., 2016; Scott et al., 1992; Sohn et al., 2018). In Asia, research specifically targeting leaf mining is limited, with a single study reporting the oldest known mine trace in South Korea (Imada et al., 2022). In South Asia, particularly India, there is an abundance of fossil leaf deposits and studies on plant‐insect interactions (e.g., Banerjee & Bera, 1998; Chandra & Singh, 1996; Khan et al., 2014, 2015; Pant & Srivastava, 1995; Singh et al., 2015; Srivastava, 1998; Srivastava & Agnihotri, 2011; Srivastava et al., 2000; Srivastava & Srivastava, 1998). Despite this extensive dataset, leaf mining is less represented and understudied, suggesting that our understanding of these interactions as preserved in the fossil record remains incomplete.

In the present study, we report and describe mine traces on fossil leaves from a Pliocene leaf assemblage of Jharkhand, eastern India deposited under a warm temperate to tropical climate with low precipitation seasonality. The objectives of our study were (i) to document ancient plant‐herbivore associations in a Pliocene forest ecosystem that is fairly similar to modern forests in this region, (ii) to discuss richness and abundance patterns of mining and galling in the Pliocene forest in the light of modern ecological studies and (iii) to investigate the ecological and evolutionary history of the terrestrial ecosystem of the Chotanagpur Plateau.

## 2 MATERIALS AND METHODS

**FIGURE 1:**
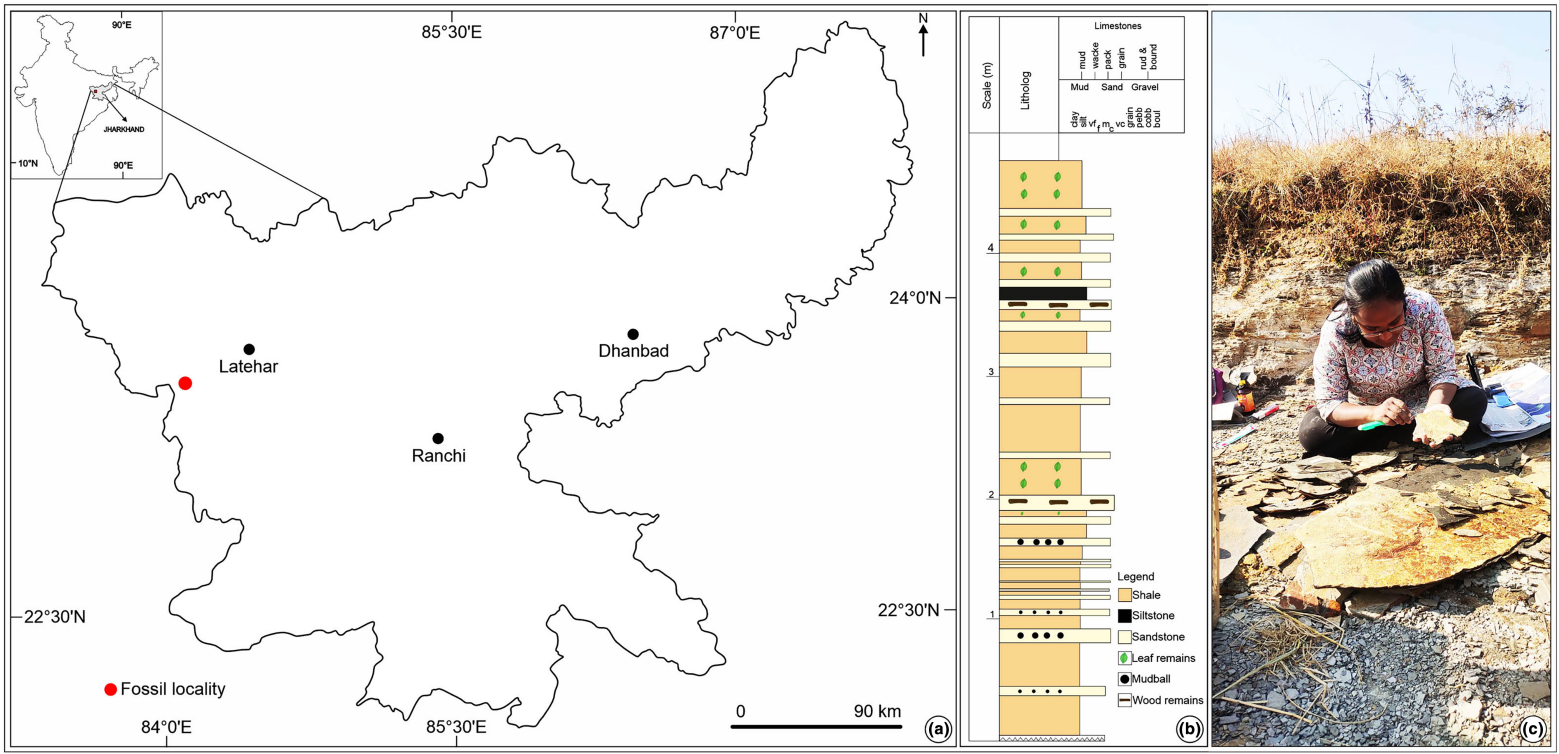
Map of the study site and lithology. (a) Map indicating the location of the studied palaeoflora (red circle). (b) A generalized lithostratigraphic column of the studied section. (c) A view of the fossiliferous locality.

Fossil leaves containing leaf extraction were collected during paleobotanical fieldwork carried out from 2019 to 2022 (Table S1). The leaves were recovered from river‐cutting sections of the latest Neogene (Pliocene: Rajdanda Formation) sediments of Mahuadanr Valley (23.3965° N, 84.1066° E; altitude 353 m a.s.l.) of Jharkhand, Chotanagpur Plateau, eastern India (Figure 1). The lithology of the studied section includes mainly shale and sandstone. Armored mud balls of rounded to elliptical shapes with coated sand grains are embedded in this sandstone unit. The upper 0.5 m of the sedimentary unit studied is composed of shale and is highly fossiliferous with an abundant fossil biota including impressions and compressions of angiosperm foliage, fruits, and flowers, fish and bird remains, insects, and carbonized wood fragments. The excellent preservation of fossil plant and animal remains suggests a reducing condition during the time of deposition. Clusters of euhedral and anhedral pyrite within the fossiliferous shale layer also indicate the presence of a reducing lacustrine environment with fluvial incursions (possibly during flood events) (Bajpai et al., 2001; Kumar et al., 2000). Previous studies considered the age of the Rajdanda Formation to be ‘late Tertiary’ (latest Neogene: Pliocene; Hazra et al., 2020; Prakash et al., 1987; Srivastava & Bande, 1992) based on lithostratigraphic correlation, plant macrofossils, and dispersed spores and pollen.

**FIGURE 2:**
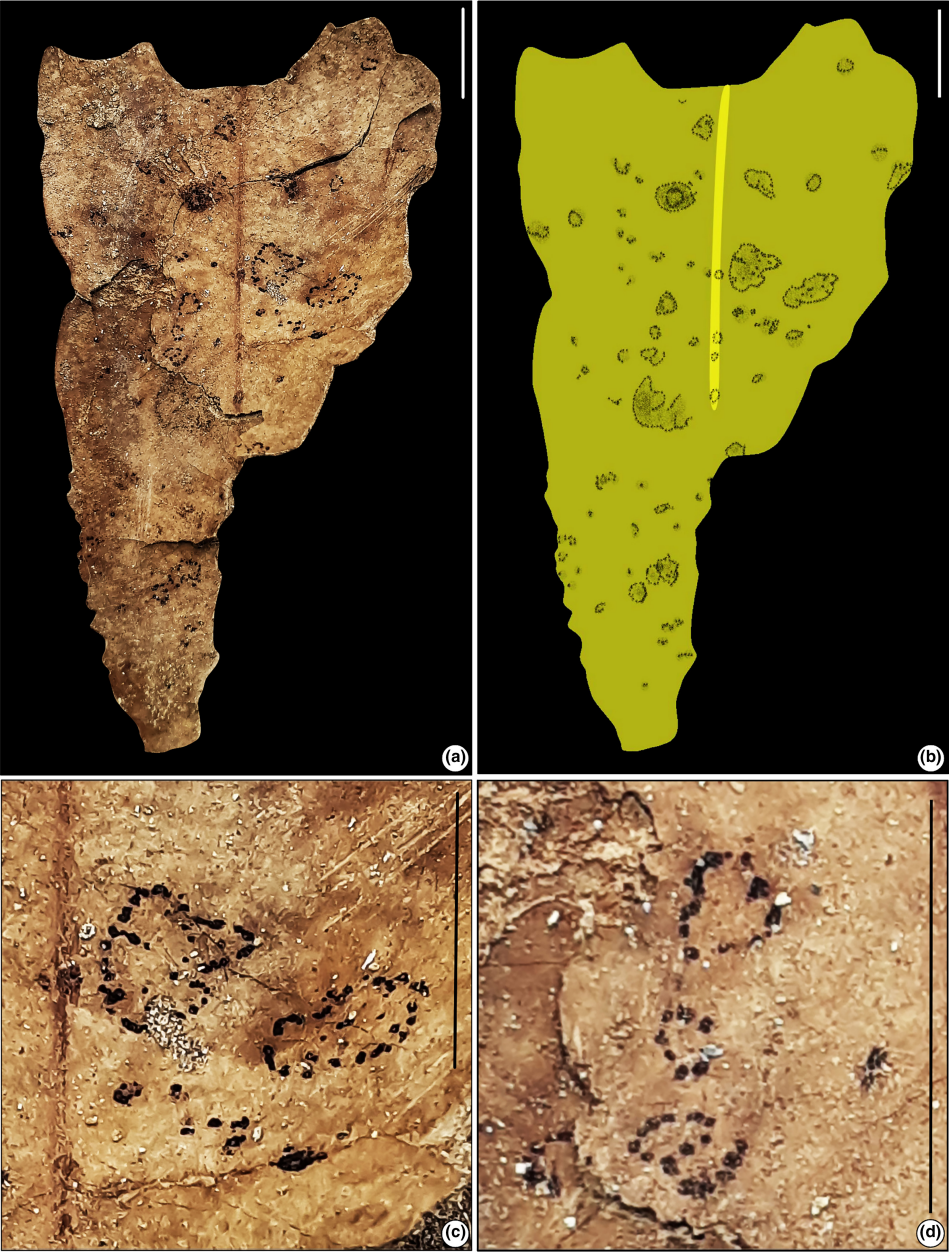
Fragments of fossil leaf with mine (SKBUH/PPL/JH/230). (a) Circular mine with particulate fecal pellets (DT 66). (b) Line drawing of the fossil specimen. (c and d) Details of mines showing the coiled structure. Scale bar = 1 cm.

**FIGURE 3:**
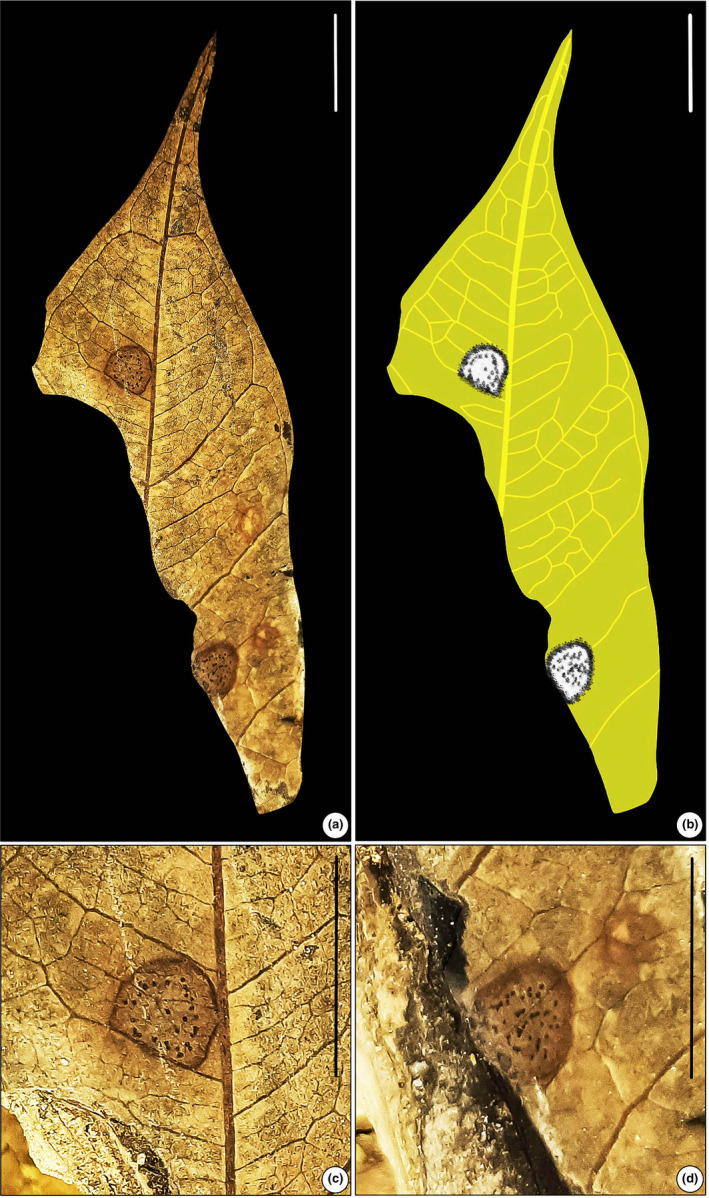
Leaf mining on *Ficus* sp. (SKBUH/PPL/JH/231). (a) Circular blotch mine with dispersed coprolites (DT 69). (b) Line drawing of the fossil specimen. (c and d) Details of mines showing circular blotch. Scale bar = 1 cm.

**FIGURE 4:**
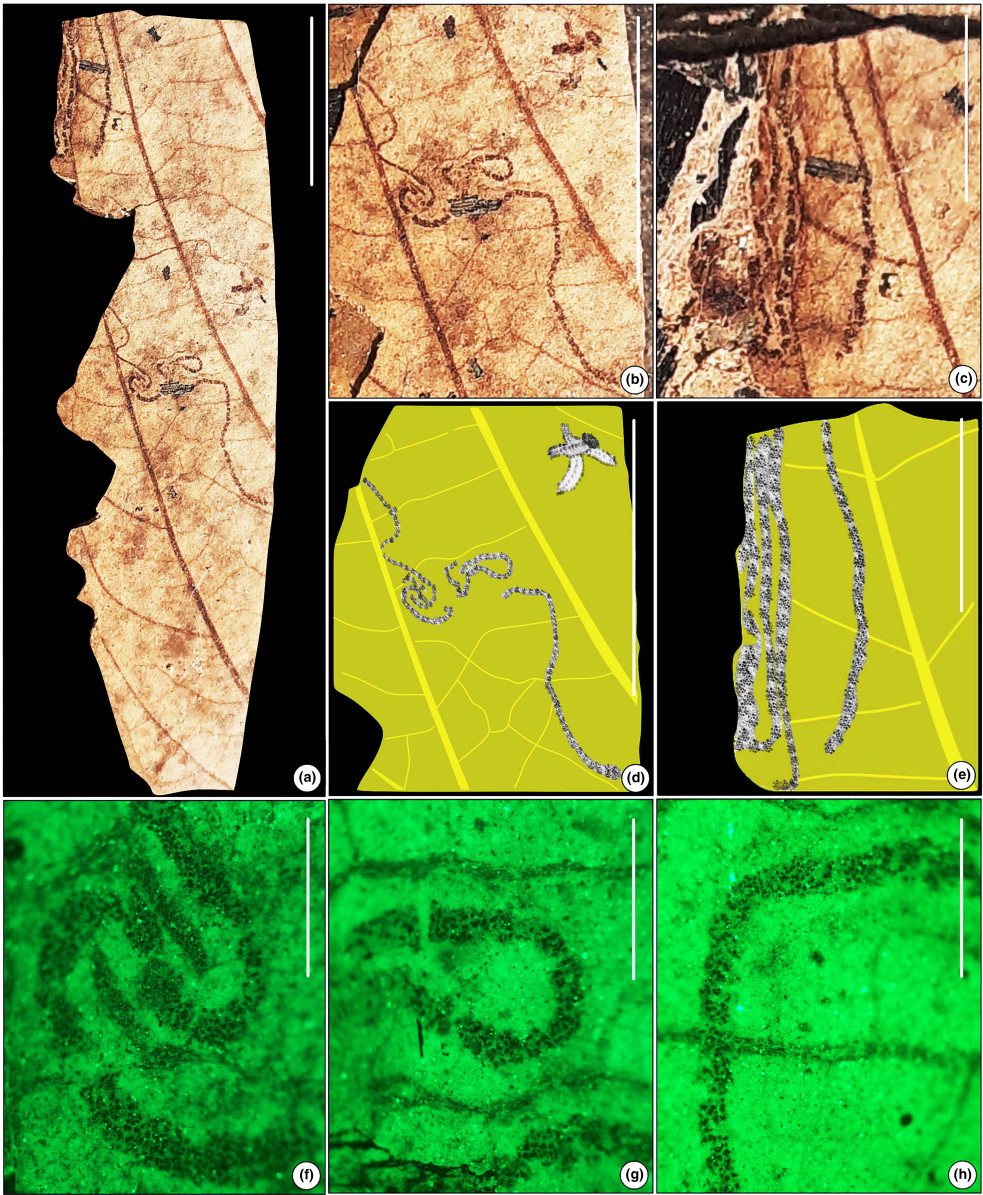
Sinusoidal mine (SKBUH/PPL/JH/232). (a) Long sinusoidal, hairline trail near the leaf margin (DT 104). (b and c) Details of mines show a sinusoidal, hairline trail. (d) Line drawing of (b). (e) Line drawing of (c). (f–h) Inverted fluorescence microscopy images of the leaf mining showing a sinusoidal structure packed with frass pellets. Scale bar = 1 cm for a, b, and d; 0.5 cm for e and c; 1 mm for f, g, and h.

**FIGURE 5:**
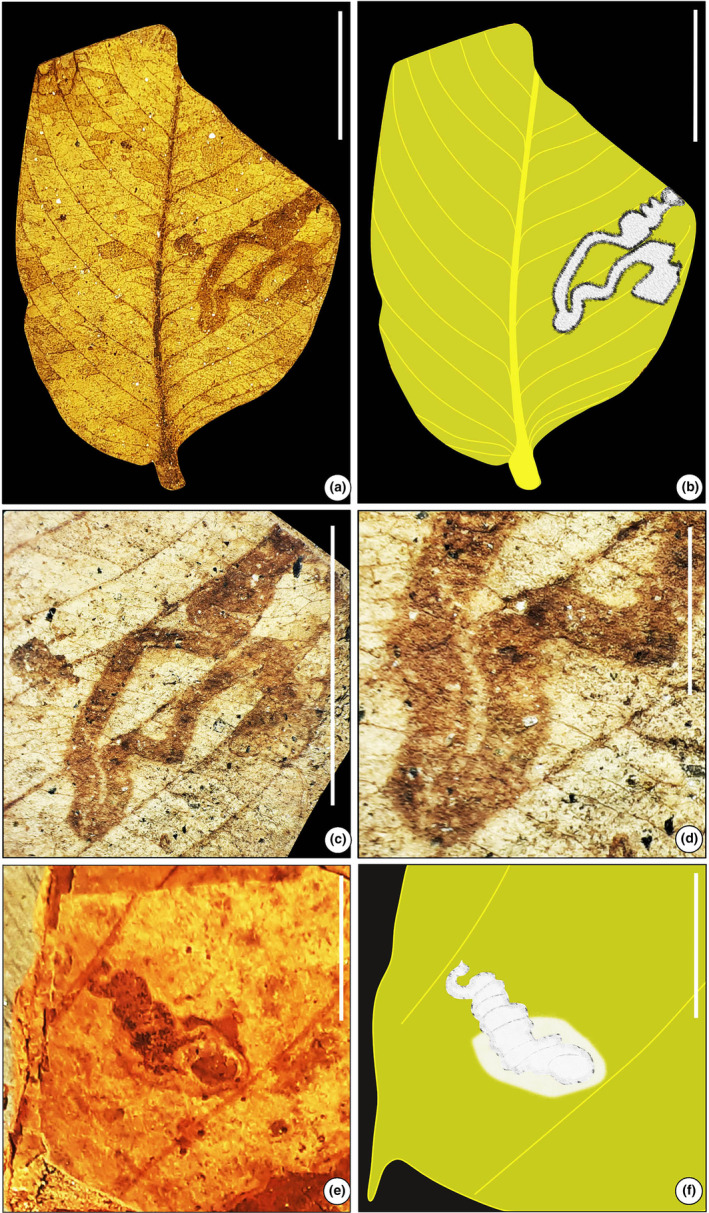
Serpentine mine (SKBUH/PPL/JH/233). (a) Robust serpentine mine, near the leaf margin (DT 91). (b) Line drawing of (a). (c) Detail of mine showing serpentine structure. (d) Enlargement of (c) showing curved structure packed with frass pellets. (e) Mine with thick frass that lies within a 2° vein having a terminal bulb‐like chamber. (f) Line drawing of (e). Scale bar = 1 cm for a–c and e and f; 0.25 cm for d.

**FIGURE 6:**
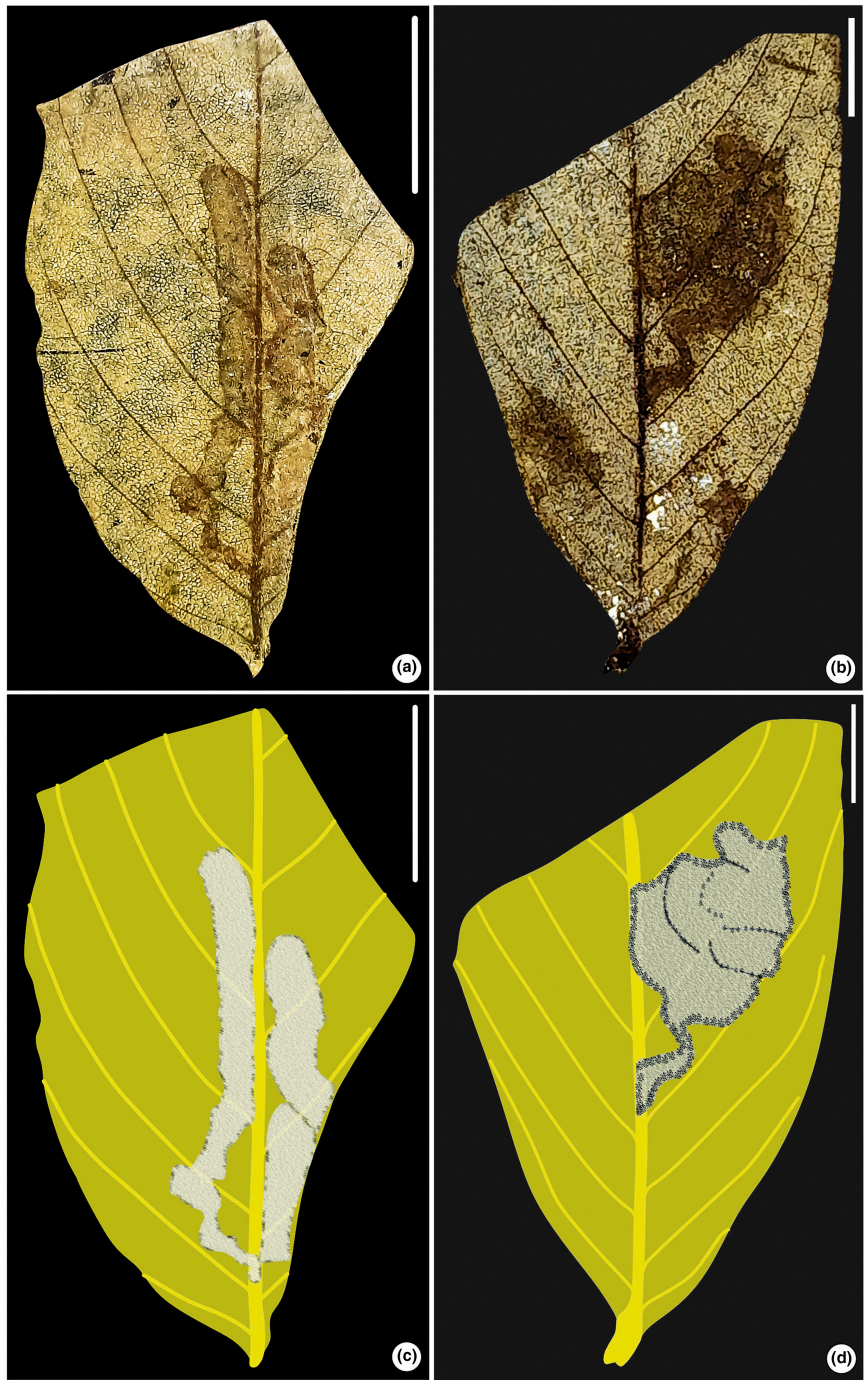
(a) Linear mine characterized by a curvilinear trajectory, ragged‐irregular margin (SKBUH/PPL/JH/282) (DT 90). (b) Line drawing of (a). (c) Robust, tortuous, wide mine (SKBUH/PPL/JH/320) (DT 91). (d) Line drawing of (c). Scale bar = 1 cm.

**FIGURE 7:**
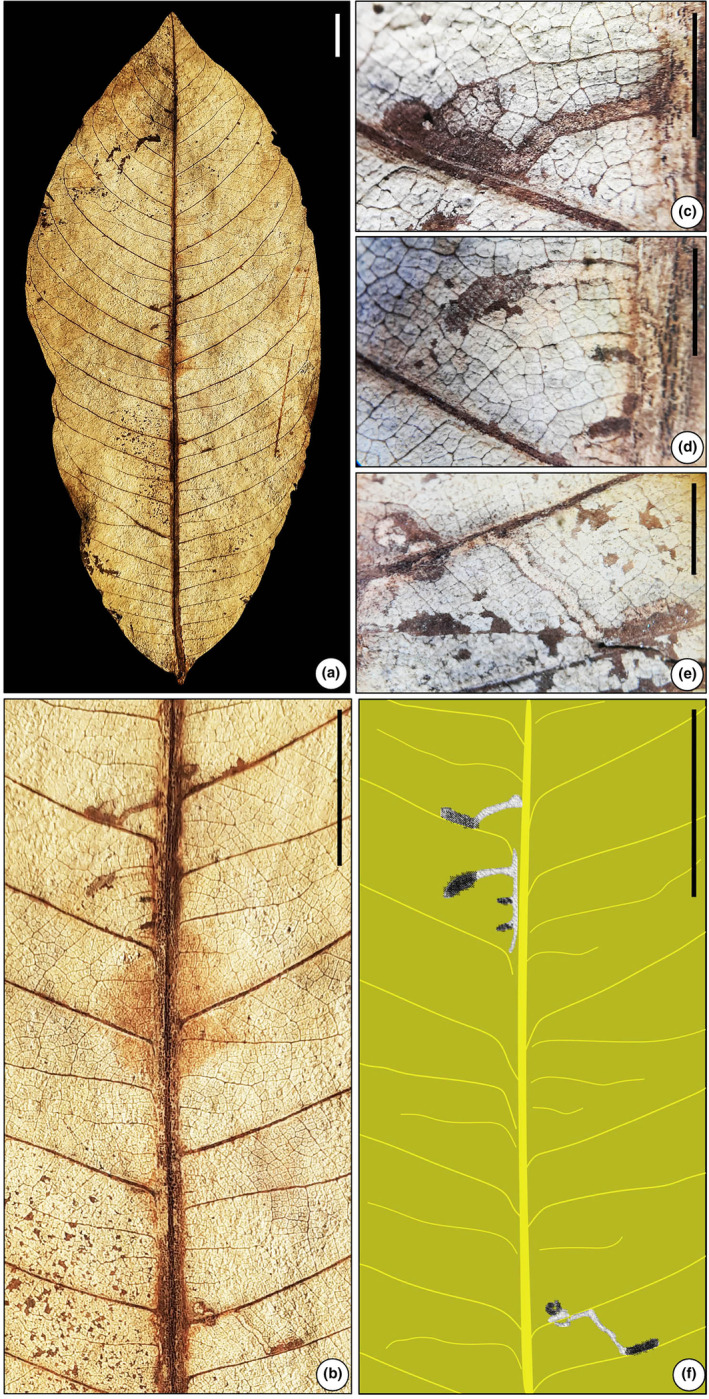
(a) Short serpentine mine on *Schleichera* leaf (SKBUH/PPL/JH/311). (b) Detail of (a). (c and d) Short serpentine mine with solid coprolites at the terminal end. (e) Short serpentine mine characterized by narrow and trajectory lines. (f) Line drawing of (b). Scale bar = 1 cm for a, b, and f; 0.25 cm for c, d, and e.

**FIGURE 8:**
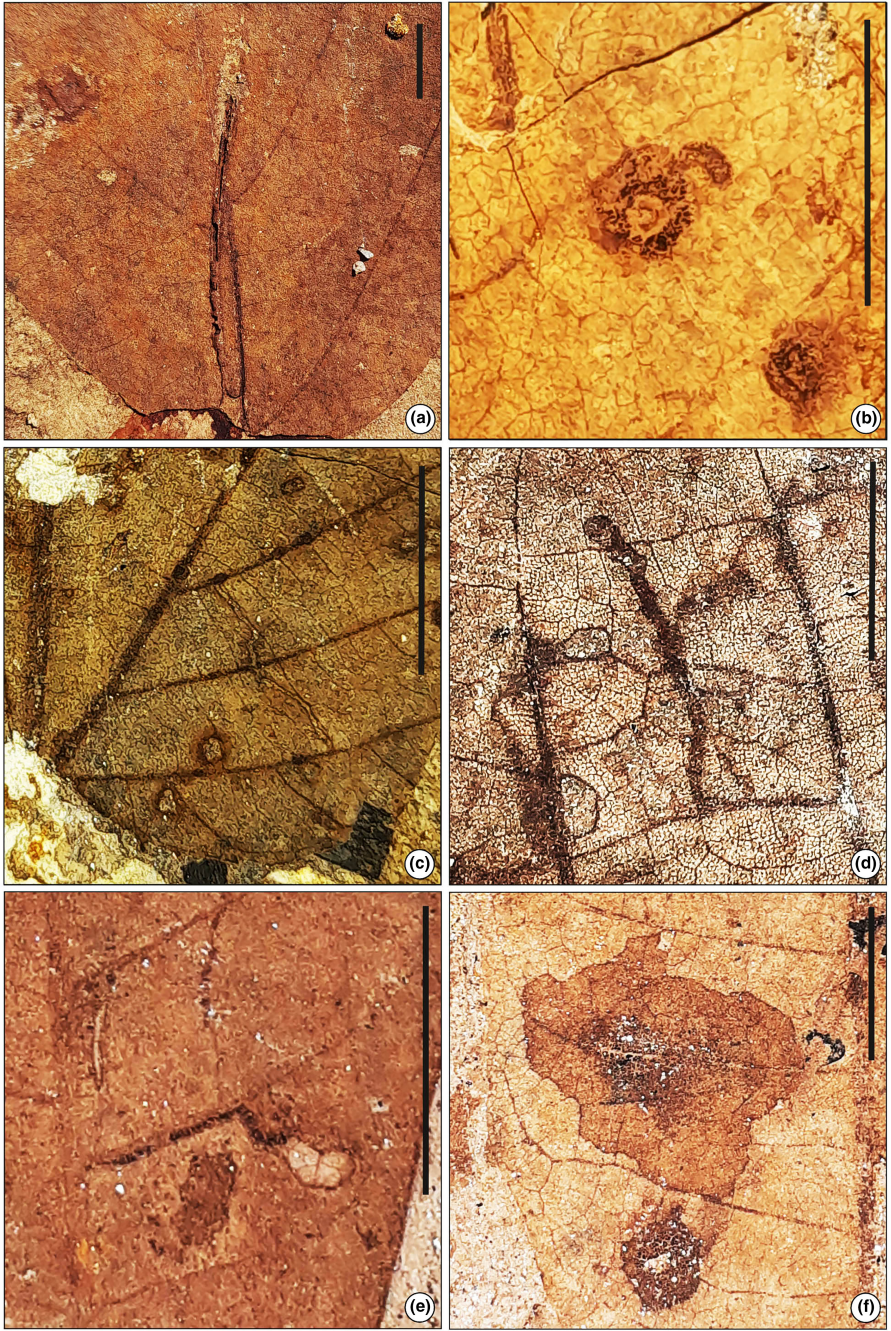
(a) Short elongate mine (SKBUH/PPL/JH/325) (DT 90). (b) Short, linear to curved early phase (SKBUH/PPL/JH/326) (DT 176). (c) Short serpentine mine with narrow trajectory limited by a primary vein (SKBUH/PPL/JH/328) (DT 105). (d) Short serpentine mine with narrow trajectory limited by a primary vein (SKBUH/PPL/JH/329) (DT 105). (e) Short serpentine mine located adjacent to primary vein (SKBUH/PPL/JH/277) (DT 111). (f) Circular blotch mine (SKBUH/PPL/JH/278) (DT 35). Scale bar = 1 cm.

**FIGURE 9:**
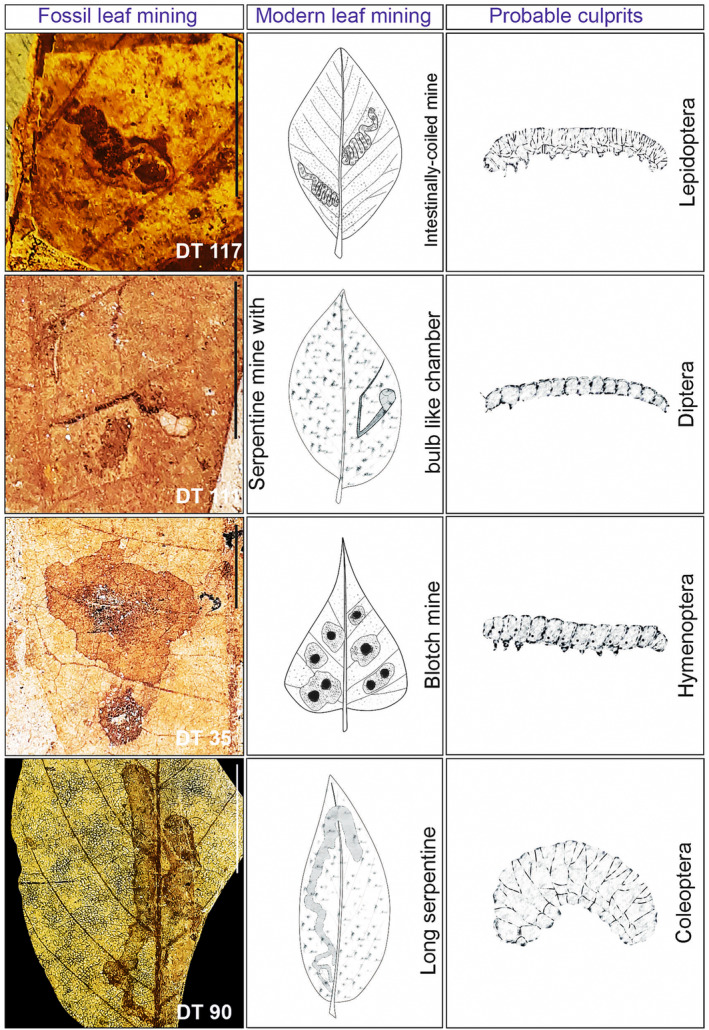
Leaf‐mining and larvae of leaf‐mining insects from four orders (Lepidoptera, Diptera Hymenoptera, and Coleoptera).

The fossil leaves (Figures 2, 3, 4, 5, 6, 7, 8) required little preparation before photography. The overlying matrix was removed with the help of fine needles, scalpels, and brushes. After cleaning, photographs were taken using a digital camera (Canon EOS 1500D), and a stereo zoom microscope, and edited with CorelDraw and Adobe Photoshop software (Figures 2, 3, 4, 5, 6, 7, 8). Details of leaf mining were drawn using CorelDraw vers. 2021 (Figures 2, 3, 4, 5, 6, 7, 8, 9). For the identification of damage patterns, we follow the guidebook, *Guide to Insect (and Other) Damage Types in Compressed Plant Fossils*, by Labandeira et al. (2007).

**FIGURE 10:**
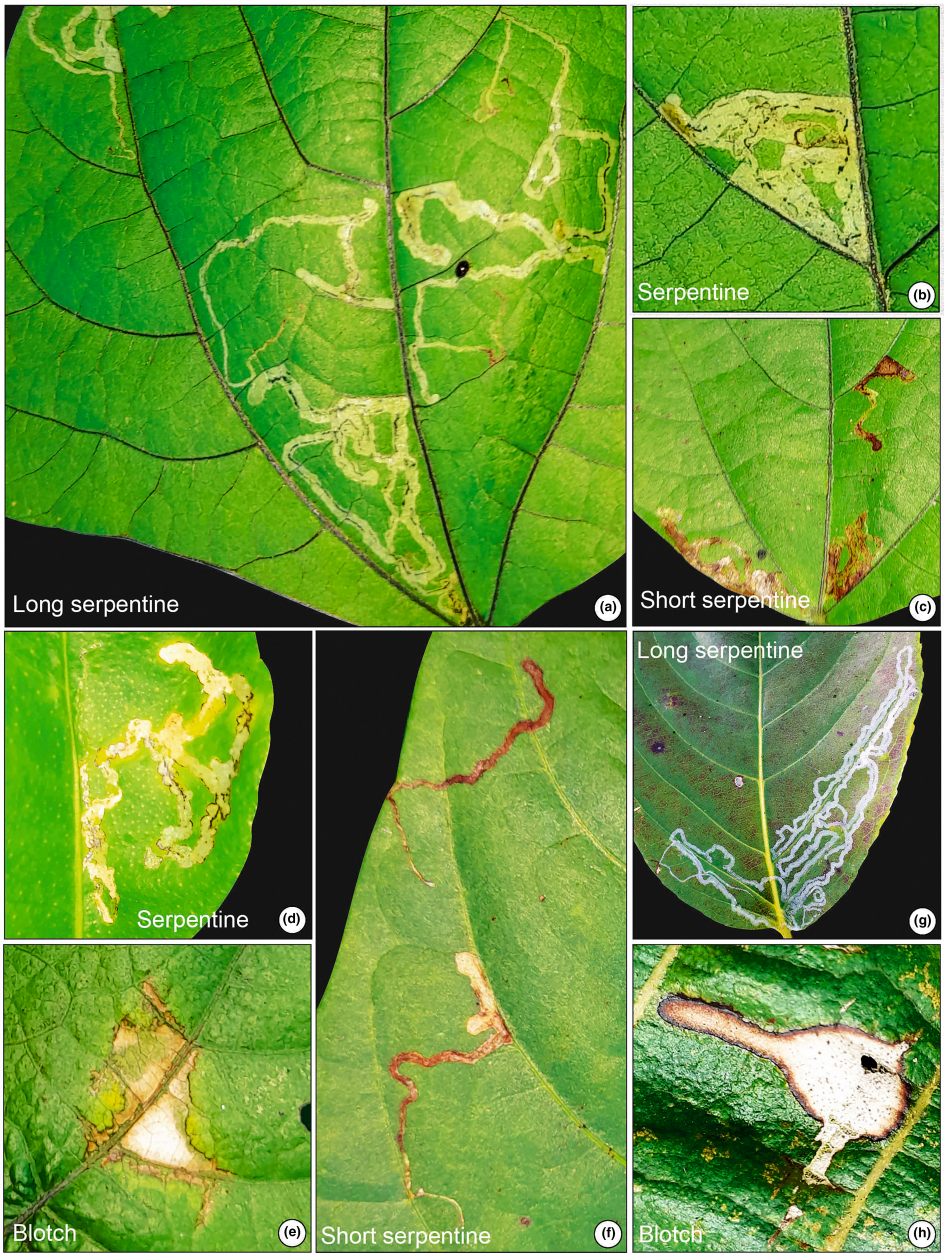
Modern leaves show various mining damage patterns collected from modern forests near the fossil locality. (a‐c), (e and f) *Phaseolus lunatus* L. (Fabaceae); (d) *Citrus limon* L. (Rutaceae); (g) *Lagerstroemia* sp. L. (Lythraceae); (h) *Tectona grandis* L. (Lamiaceae).

We briefly observed the surrounding modern‐day forests near the fossil exposures and noted some similar mining types on contemporary leaves, of which we took a few photographs (Figure 10). We followed the terminologies of Hering (1951) for the general description of leaf mining. The fossil specimens reported in this manuscript are housed in the Department of Botany, Palaeobotany and Palynology Laboratory, Sidho‐Kanho‐Birsha University, Purulia, India.

## 3 RESULTS

Investigating 1088 fossilized leaves, we identified 11 distinct mining morphotypes (Table 1, Figures 2, 3, 4, 5, 6, 7, 8), which were observed on 21 leaves representing 11 plant families. These families were represented by 875 leaves in total, with Dipterocarpaceae, Fabaceae, Lauraceae, and Moraceae showing the highest frequency of mines (Table S1). In modern dry tropical deciduous forests of the Chotanagpur Plateau, Dipterocarpaceae and Fabaceae are typically represented by semi‐deciduous trees, which shed their leaves in the dry season. Each identified morphotype closely resembles standardized damage types (DTs) as outlined by Labandeira et al. (2007).

**TABLE 1:** List of plant hosts related to the different damage types (DT) identified.

Host plants	DT reference	Number of leaves concerned
Myrtaceae, Unidentified dicot leaf	DT 66	2
Moraceae, Lauraceae, Combretaceae	DT 69	3
Moraceae, Anacardiaceae, Unidentified dicot leaf	DT 104	3
Rhamnaceae, Myrtaceae, Fababceae	DT 91	3
Malvaceae, Fababceae, Unidentified dicot leaf	DT 117	3
Unidentified dicot leaf	DT 111	1
Apocynaceae, Unidentified dicot leaf	DT 35	2
Dipterocarpaceae, Rhamnaceae	DT 42	2
Lauraceae, Sapindaceae, Lauraceae	DT 105	3
Dipterocarpaceae, Moraceae	DT 176	2
Dipterocarpaceae, Fabaceae	DT 131	2

### 3.1 Description of leaf mines

#### 3.1.1 Leaf‐mining type 1 (Figure 2)

Mine circular, ca. 2–6 mm in diameter, characterized by a coiled middle portion, almost evenly‐spaced particulate fecal pellets forming a circular coiled structure; distributed throughout the lamina; fecal pellets distributed throughout the mine, composed of spheroid pellets measuring ca. 0.1 mm in diameter followed by smaller fragments of pellets.

Damage type: DT 66
Host: Unidentified dicot leaf
Specimen no.: SKBUH/PPL/JH/230
Inferred order: Diptera

#### 3.1.2 Leaf‐mining type 2 (Figure 3)

Blotch mine, typically circular or slightly oval in shape, featuring dispersed coprolites surrounded by a pronounced reaction rim. The outer edge of the mine is notably thickened and darkened in color. The mines are ca. 4–5 mm in diameter, although the fossil leaf specimen containing the mine is broken in the basal region. The mines are located between two secondary veins, avoiding the main leaf veins and the leaf margin.

Damage type: DT 69
Probable host: *Ficus* sp. (Moraceae)
Specimen no.: SKBUH/PPL/JH/231
Inferred order: Lepidoptera

#### 3.1.3 Leaf‐mining type 3 (Figure 4)

Wavy and intersecting narrow trail; mine begins near the leaf edge, runs between secondary veins without crossing the central vein, and cuts across smaller veins; thin lines between waste clusters. (NB: partial remains of Diptera on the leaf surface).

Damage type: DT 104
Host: Unidentified dicot leaf
Specimen no.: SKBUH/PPL/JH/232
Inferred order: Hymenoptera

#### 3.1.4 Leaf‐mining type 4 (Figure 5a–d)

Robust serpentine mine; no pellets; shape intestine‐like (Figure 5c, upper part of panel), becoming serpentine (Figure 5c, lower part, Figure 5d), ca. 20–30 mm long and ca 1 mm wide; characterized by several turns, a trajectory limited by secondary and tertiary veins. Mine ending in an elliptical terminal chamber (Figure 5a,b). The mine tracks the tertiary veins until it hits a secondary vein, then turns sharply and follows a wavy path; it can also cross a secondary vein.

Damage type: DT 91
Probable hosts: *Berhamniphyllum* (Rhamnaceae)
Specimen no.: SKBUH/PPL/JH/233
Inferred order: Lepidoptera

#### 3.1.5 Leaf‐mining type 5 (Figure 5e,f)

Short serpentine mine, coiled, ca. 2–3 mm thick with dense coprolites; located near the main vein between the center and edge, then moves towards the secondary veins. The mine's final stages are within the secondary vein, ending in a bulb‐like chamber with thin tissue and no coprolites.

Damage type: DT 117
Host: Unidentified dicot leaf
Specimen no.: SKBUH/PPL/JH/284
Inferred order: Lepidoptera

#### 3.1.6 Leaf‐mining type 6 (Figure 6)

Incomplete serpentine mine, about 20–30 mm long, with thin tissue and serrated edges. The mine starts near the primary vein and curves along the primary vein and towards the edge.

Damage type: DT 42
Probable hosts: *Berhamniphyllum* (Rhamnaceae)
Specimen no.: SKBUH/PPL/JH/282
Inferred order: Lepidoptera

#### 3.1.7 Leaf‐mining type 7 (Figures 7 and 8c,d)

The mine displays a serpentine pattern, measuring less than 10 mm in length. It begins with a narrow trajectory of 0.3 mm adjacent to a primary vein, near the leaf's center. As it progresses, the mine quickly expands in width, ca. 1–1.5 mm. It ends in a rounded terminal chamber approximately 2.65 mm in size.

Damage type: DT 105
Probable host: *Schleichera* sp. (Sapindaceae), Fabaceae
Specimen no.: SKBUH/PPL/JH/311; SKBUH/PPL/JH/323; SKBUH/PPL/JH/324
Inferred order: Diptera

#### 3.1.8 Leaf‐mining type 8 (Figure 8a)

Short extended mine with spindle‐shaped markings on the leaf. Starting at a width of around 0.5 mm, the mine gradually widens to 3–4 mm.

Damage type: DT 131
Probable host: *Grewia* sp., Fabaceae
Specimen no.: SKBUH/PPL/JH/325
Inferred order: Diptera

#### 3.1.9 Leaf‐mining type 9 (Figure 8b)

Mine short, linear to curved early phase, ca. 2 mm long, characterized by much expanded ovoidal to circular terminal phase.

Damage type: DT 176
Probable host: *Ficus* sp. (Moraceae)
Specimen no.: SKBUH/PPL/JH/326
Inferred order: Diptera

#### 3.1.10 Leaf‐mining type 10 (Figure 8e)

It is a short serpentine mine, ca 10 mm with thick frass. The mine starts near the primary vein or centrally between the mid‐vein and edge, then extends towards the edge, staying between two secondary veins. The mine ends in a bulb‐like chamber with thin, skeletonized tissue.

Damage type: DT 111
Host: Unidentified dicot leaf
Specimen no.: SKBUH/PPL/JH/277
Inferred order: Diptera

#### 3.1.11 Leaf‐mining type 11 (Figure 8f)

A circular blotch mine located between two secondary veins, partly forming a border with one secondary vein, but without crossing it. Leaf‐mining diameter measuring ca. 20 mm. Coprolites grouped inside the mine in a circular shape less than 10 mm in diameter, almost in the center of the gallery.

Damage type: DT 35
Host: Unidentified dicot leaf
Specimen no.: SKBUH/PPL/JH/278
Inferred order: Diptera

## 4 DISCUSSION

### 4.1 Differences in modern climate and inferred Pliocene climate in eastern India

The modern climate in Jharkhand is a typical monsoon climate, ranging from temperate *Cw* to tropical *Aw* climate types according to the Köppen‐Geiger climate classification (Peel et al., 2007). At present, the ratio between the three consecutive wettest (P3wet) and driest (P3dry) months amounts to ca. 20. In contrast, if taking the inferred climate parameters for the Pliocene at face value (Hazra et al., 2020), the precipitation seasonality in the Pliocene would have been much weaker, the ratio of P3wet to P3dry being between 3 and 4 (Table S2). Together with the inferred temperature climate parameters, the climate in the Mahuadanr valley would have been at the transition between a humid warm temperate Cf and a moist tropical *Am* climate.

Considering previous investigations about factors determining mining and galling richness such as the ‘habitat mediated richness hypothesis’ of Fernandes et al. (2004) and the ‘harsh environment hypothesis’ of Price et al. (1987), we would expect increased mining and relatively lower galling in the Pliocene forest of Jharkhand. Indeed, our results are to some part contrary to this expected trend; although galling is less rich than mining, it is much more frequent in our fossil leaf assemblage (Table S1). Our findings, highlighting a discrepancy between expected and observed patterns of mining and galling in the Pliocene forest, resonate with contemporary studies indicating the absence of a universal pattern in plant‐insect interactions. For instance, previous research in the Cerrado of Brazil vegetation found leaf‐mining insects more commonly in mesic than xeric habitats, contrary to expectations (Fernandes et al., 2004). Similarly, studies across Australia's precipitation gradients observed no straightforward correlation between rainfall and the prevalence of leaf miners, with leaf morphology, rather than climate, showing a stronger link to mining activity (Sinclair & Hughes, 2008). In the realm of galling, while some studies posited a preference for seasonal and xeric environments (Fernandes & Lara, 1993; Fernandes & Price, 1992; Marques et al., 2000), others found no clear evidence that climate alone dictates gall‐inducing insect richness, suggesting instead factors like soil fertility could play a more significant role (Blanche, 2000; Blanche & Ludwig, 2001). Furthermore, the exceptional richness of gall‐inducing insects in the Amazonian rainforest canopies (Júlião et al., 2014) underscores the complexity of these interactions, challenging the notion of predictable patterns based solely on climatic conditions. These examples, mirroring our own observations, underscore the multifaceted nature of plant‐insect relationships, influenced by a mosaic of environmental factors.

### 4.2 Possible environmental influence on leaf‐mining diversity

Moister environments and softer, larger leaves are associated with higher mining diversity (Andrew & Hughes, 2005; Fernandes et al., 2004; Sinclair & Hughes, 2008) in tropical savannahs of Australia and South America. The miners preferentially select the largest leaves for oviposition (Faeth et al., 1981; Sinclair & Hughes, 2008). Aside from the thickness and size of the leaves, the increasing mining richness observed in the tropics might be linked to increased leaf water content and relative humidity (Scriber, 1977). Similar conditions can be inferred from the leaf‐mining documented here and the overall high levels of plant‐arthropod interactions in the Pliocene tropical forests of the Chotanagpur Plateau. Notably, leaf‐mining morphotypes had a relatively high richness but very low abundance. This might indicate, that mining insects did not meet optimal conditions in the paleoforest of the Chotanagpur Plateau. One reason for this could be that the dominating tree species of the Dipterocarpaceae and Fabaceae might have been semideciduous trees with rather hard leaf texture. These plants would have favored gall‐inducing over mining insects. In contrast, more mesophyllic leaves suitable for mining insects may have been relatively rare in these forests. Based on similarities in the form, size, shape, and position of the mines in the fossil leaves and extant plants, the responsible culprits were inferred (cf. Figures 9 and 10).

### 4.3 Forest composition and patterns of mining and galling in fossil assemblages

The Mahuadanr Valley leaf assemblage is dominated by Fabaceae (Table S1). This is complemented by the presence of Anacardiaceae, Moraceae, Dipterocarpaceae, and other families such as Rhamnaceae and Rubiaceae. Notably, the composition of these ancient forests mirrors the current regional vegetation, as highlighted in previous paleobotanical studies (Singh & Prasad, 2009). Today, the forests in Jharkhand are categorized as northern tropical dry deciduous forests (Champion & Seth, 1968). These forests exhibit a deciduous canopy, prominently featuring trees like *Shorea robusta* C.F. Gaertn., Dipterocarpaceae. While some forests have a dominant presence of Fabaceae, others host a diverse mix of families including Anacardiaceae, Rubiaceae, and Combretaceae.

Our reassessment of arthropod insect damage types in the Pliocene leaf assemblage of Mahuadanr Valley confirmed a previous study focused on leaf galls (Hazra et al., 2022), which found that galling was most common in the fossil leaf assemblage (Table S1[5]). Mining was most common in Dipterocarpaceae, Fabaceae, Moraceae, and Lauraceae, and least common in Rhamnaceae, Myrtaceae, Anacardiaceae, and Malvaceae. Overall, mining was much less common in the leaf assemblage than galling. However, similar to the pattern seen in mining, galling was most common in Fabaceae, Anacardiaceae, Moraceae, Combretaceae, and Rhamnaceae, followed by Dipterocarpaceae, Malvaceae, and others. The three most galled families are also the most common in the overall leaf fossil record. This would support the ‘habitat mediated richness hypothesis’ of Fernandes et al. (2004). Interestingly, while nearly all the taxa mentioned here are typical elements of modern tropical deciduous forests in the region, Lauraceae are not common in these forests. In addition, the fossil leaf assemblage studied here may derive from more than a single habitat. Whereas well‐drained stands with Fabaceae or Dipterocarpaceae dominated forest might have been more exposed to galling, riparian forest with more mesic vegetation could have accounted for the mining observed in our material. Similar observations are known from modern ecological studies. For example, Cuevas‐Reyes et al. (2004) reported a higher abundance of galling in well‐drained habitats of tropical dry forest in Mexico than in riparian stands.

### 4.4 Inferred paleoclimate and insect‐plant interactions

The leaf physiognomy of the rich leaf assemblage from Mahuadanr Valley points to absence or a weaker monsoon during the Pliocene as compared to the present (Table S2; Hazra et al., 2020). Nevertheless, the highly abundant and diverse record of galling would indicate that galling insects were favored by semideciduous, hard‐leaved trees (for instance, species‐rich Fabaceae) typical of the Pliocene tropical deciduous forests. This situation compares well with modern observations from South American tropical forests thriving under a fully humid tropical rainforest climate (*Af* Köppen‐Geiger climate type; Peel et al., 2007), where a markedly higher diversity of galling was observed in the upper canopy layer, where leaves tend to be more sclerophyllous (Júlião et al., 2014). In addition, the weak precipitation seasonality without distinct dry periods (Table S2) would allow for some mining on the diverse deciduous tree flora of more mesic strata of the forest.

Observations in modern temperate deciduous forests suggested that leaf mining is more common in dominant species than in rare ones and that the diversity of leaf‐mining insects is greater between the host plant families with the highest number of species (Dai et al., 2018). A high number of species within a family would increase the availability of leaf‐mining niches. Our results are difficult to compare to these modern observations, as the most abundant leaf types in a fossil assemblage may not represent the most abundant species of the past vegetation due to taphonomic biases. For instance, the differential preservation of tougher leaves over more delicate ones, which decompose more readily, could skew our understanding of past plant diversity and insect‐plant interactions (e.g., Retallack, 2019). Moreover, modern studies suggest that in a given forest ecosystem, riparian habitats tend to be less rich in galling as compared to well‐drained habitats (e.g., Cuevas‐Reyes et al., 2004; Fernandes & Price, 1992). In contrast, riparian forests would be relatively richer in mining (Hespenheide, 1991). In fossil leaf assemblages, riparian habitats may be overrepresented as they are located next to the depositional area (Spicer, 1991). Nevertheless, we note that mining is generally rare; the ratio of mined to total number of leaves per taxon is less than 1:20 except for in Lauraceae, Myrtaceae, and Sapindaceae (Table S1[4]). Within the family with the highest representation among leaf remains (227 instances, Fabaceae), only three leaves were found to be mined. In contrast, 25 to more than 50% of all leaves per family are galled. Our observations imply that the relationship between plant diversity and leaf‐mining insect diversity, as reported by Dai et al. (2018) for the transition of temperate forest to grassland in Inner Mongolia, may not manifest in the same manner in tropical deciduous forests, where galling seems to be more prevalent. Detailed actuopaleontological observations in these forests including drier and moister variants (Champion & Seth, 1968) are needed to better understand the observed patterns of galling and mining diversity.

### 4.5 Continuity of insect‐host plant association

A preliminary examination of the extant tropical forests adjacent to the fossil outcrops revealed that similar types of feeding traces were present on modern leaves from different angiosperm taxa (Figures 9 and 10). Based on our investigation, culprits that left their traces in the fossil leaf assemblage might belong to four insect orders: Lepidoptera (moths), Diptera (flies), Coleoptera (beetles), and Hymenoptera (sawflies) causing similar damage traces on modern leaves of several angiosperm taxa (Figures 9 and 10). Overall, this would suggest no marked change in leaf‐feeding strategies of this type of herbivorous insect since the Pliocene. Recent studies also suggest that the same damage inducers (i.e. Lepidoptera, Diptera, Coleoptera, and Hymenoptera) were active in the tropical forest of the Mahuadanr Valley on the Chotanagpur Plateau, eastern India (Hazra et al., 2020; Shukla et al., 2000).

Apart from climate, other factors such as dominating plant families and phylogenetic signal (Dai et al., 2018), low soil fertility (Blanche & Ludwig, 2001), or habitat heterogeneity (Cuevas‐Reyes et al., 2004) may have determined the observed patterns of specialized plant‐insect interaction during the Pliocene. In addition, a specific feature of the here investigated leaf assemblage and the modern forest in the area may be the high amount of deciduous leaves with hard texture. Regardless of the macroclimate, insect leaf‐mining communities have higher species richness in more mesic sites because of the availability of suitable leaf types (softer, larger leaves) compared to more arid ones (thicker, more sclerophyllous leaves) (Andrew & Hughes, 2005; Fernandes et al., 2004; Sinclair & Hughes, 2008). The observed increase in mining richness within tropical regions (Hespenheide, 1991) may be linked to heightened leaf water content and ambient humidity, which are conducive to larval development and overall insect fitness (Connor & Taverner, 1997). Additionally, growth rates and pupal development are negatively impacted by low leaf water and relative humidity, as highlighted by Scriber (1977). Thus, low relative humidity reduces fitness by limiting the richness of leaf mining in arid areas. The richness of mining damage types observed in the Pliocene leaf assemblage from the Mahuadanr Valley along with their low abundance might reflect suboptimal growing conditions for leaf mining in a weakly seasonal climate.

However, it is important to reiterate that few studies have indicated that galling intensity may not correlate directly with aridity but could instead be more closely related to plant species (Blanche, 2000; Veldtman & McGeoch, 2008). Furthermore, research by Bairstow et al. (2010) suggests that both plant traits and climatic factors contribute to the diversity of leaf miners and gallers, emphasizing a complex interplay between host plant characteristics and environmental conditions. This nuanced perspective underscores the need for caution in interpreting fossil records in relation to present‐day ecological patterns. It is through modern analyses using methods that are replicable in the fossil record that we may significantly advance our understanding of plant‐insect interaction patterns.

## 5 CONCLUSION

Our study of specialized plant‐insect interactions preserved on dispersed leaves from Pliocene strata originating from a dry deciduous tropical forest of eastern India revealed high richness of mining and galling. Whereas mining was not abundant in the fossil leaf assemblage, galling occurred on nearly half of the dispersed leaves. The inferred paleoclimate for this region was a warm temperate to tropical climate with weak precipitation seasonality. The low abundance of mining coupled with high galling intensity was somehow counterintuitive in view of the widespread notion that galling typically occurs in harsh environments. We propose that the particular leaf morphology of many constituents of the tropical dry deciduous forest of eastern India, deciduous leaves with a markedly hard leaf texture as found in many Fabaceae, may have favored galling over mining. In addition, different habitats in the same forest ecosystem, well‐drained forest, and riparian stands, may have favored different types of specialized plant–insect interactions, with miners being more common in mesic riparian environments. Many more data from modern vegetation types are needed to differentiate a number of biotic and abiotic factors determining mining and galling diversity across the globe. Paleontological studies must document patterns, which can be discussed in light of increasing insights into the nature of the interactions between endophagous insect species and their host plants.

## AUTHOR CONTRIBUTIONS

**Benjamin Adroit:** Conceptualization (lead); formal analysis (lead); writing – original draft (lead). **Taposhi Hazra:** Conceptualization (supporting); data curation (lead); writing – original draft (supporting). **Thomas Denk:** Conceptualization (lead); formal analysis (lead); writing – original draft (lead). **Subhankar Kumar Sarkar:** Conceptualization (supporting); data curation (lead). **Mahasin Ali Khan:** Conceptualization (lead); data curation (lead); formal analysis (supporting); writing – original draft (supporting).

## CONFLICT OF INTEREST STATEMENT

The authors declare no conflict of interest.

### OPEN RESEARCH BADGES

This article has earned an Open Data badge for making publicly available the digitally‐shareable data necessary to reproduce the reported results. The data is available at https://github.com/BenjaminAdroit/Leaf_mining_India_reflect_Structure_and_Climate.

## Supporting information


Table S1.

Table S2.

## Data Availability

The raw data underlying the research presented here are available in the supplementary files and can be accessed at DOI: 10.6084/m9.figshare.25075208.
